# SPOCK1 as a potential cancer prognostic marker promotes the proliferation and metastasis of gallbladder cancer cells by activating the PI3K/AKT pathway

**DOI:** 10.1186/s12943-014-0276-y

**Published:** 2015-01-27

**Authors:** Yi-Jun Shu, Hao Weng, Yuan-Yuan Ye, Yun-Ping Hu, Run-Fa Bao, Yang Cao, Xu-An Wang, Fei Zhang, Shan-Shan Xiang, Huai-Feng Li, Xiang-Song Wu, Mao-Lan Li, Lin Jiang, Wei Lu, Bao-San Han, Zhi-Gang Jie, Ying-Bin Liu

**Affiliations:** Laboratory of General Surgery and Department of General Surgery, Xinhua Hospital, Affiliated with Shanghai Jiao Tong University, School of Medicine, No. 1665 Kongjiang Road, Shanghai, 200092 China; Institute of Biliary Tract Disease, Shanghai Jiao Tong University School of Medicine, No. 1665 Kongjiang Road, Shanghai, 200092 China; The Department of General Surgery, First affiliated hospital of Nanchang University, No.17 Yongwaizheng street, Nanchang, 330006 Jiangxi China

**Keywords:** Gallbladder cancer, SPOCK1, Tumor progression, RNA interference, Epithelial-mesenchymal transition

## Abstract

**Background:**

Gallbladder cancer (GBC) is a leading cause of cancer-related death worldwide, and its prognosis remains poor, with 5-year survival of approximately 5%. In this study, we analyzed the involvement of a novel proteoglycan, *Sparc/osteonectin, cwcv, and kazal-like domains proteoglycan 1* (SPOCK1), in the tumor progression and prognosis of human GBC.

**Methods:**

SPOCK1 expression levels were measured in fresh samples and stored specimens of GBC and adjacent nontumor tissues. The effect of SPOCK1 on cell growth, DNA replication, migration and invasion were explored by Cell Counting Kit-8, colony formation, EdU retention assay, wound healing, and transwell migration assays, flow cytometric analysis, western blotting, and *in vivo* tumorigenesis and metastasis in nude mice.

**Results:**

SPOCK1 mRNA and protein levels were increased in human GBC tissues compared with those in nontumor tissues. Immunohistochemical analysis indicated that SPOCK1 levels were increased in tumors that became metastatic, compared with those that did not, which was significantly associated with histological differentiation and patients with shorter overall survival periods. Knockdown of SPOCK1 expression by lentivirus-mediated shRNA transduction resulted in significant inhibition of GBC cell growth, colony formation, DNA replication, and invasion *in vitro*. The knockdown cells also formed smaller xenografted tumors than control GBC cells in nude mice. Overexpression of SPOCK1 had the opposite effects. In addition, SPOCK1 promoted cancer cell migration and epithelial-mesenchymal transition by regulating the expression of relevant genes. We found that activation of the PI3K/Akt pathway was involved in the oncogenic functions of SPOCK1 in GBC.

**Conclusions:**

SPOCK1 activates PI3K/Akt signaling to block apoptosis and promote proliferation and metastasis by GBC cells *in vitro* and *in vivo*. Levels of SPOCK1 increase with the progression of human GBC. SPOCK1 acts as an oncogene and may be a prognostic factor or therapeutic target for patients with GBC.

**Electronic supplementary material:**

The online version of this article (doi:10.1186/s12943-014-0276-y) contains supplementary material, which is available to authorized users.

## Background

Gallbladder cancer (GBC) is the most common biliary tract malignancy and the seventh most common gastrointestinal cancer [1]. The Surveillance, Epidemiology, and End Results (SEER) program estimates the incidence of GBC at 2.5 cases per 1 × 10^5^ people. Despite the relatively low incidence rate, GBC-associated mortality is higher than that of other cancers [2]. The prognosis of advanced gallbladder carcinoma is very poor, and the 5-year survival rate is only approximately 5% [3]. This poor survival rate is because of the early spread of tumors via lymphatic, perineural, and hematogenous routes as well as direct invasion into the liver [4]. Therefore, patient prognoses may be improved by identifying novel and effective therapeutic targets for the treatment of this disease and increasing our understanding of biomarkers that can predict therapeutic responses.

The human genome sequencing project has found that 70% of the genome is transcribed, but only up to 2% of the human genome serves as blueprints for proteins [5,6]. One oncogene, *sparc/osteonectin, cwcv, and kazal-like domains proteoglycan 1* (*SPOCK1*), has been found to play a critical role in cell-cycle control, apoptosis, DNA repair, and metastasis [7]. SPOCK1 encodes a matricellular glycoprotein belonging to a novel Ca^2+^-binding proteoglycan family. Members of this protein family, which share a similar N-terminus, follistatin-like domain, and C-terminus, are involved in cell proliferation, adhesion, and migration [8]. Other members of this family include SPARC, testican-2, and testican-3. Among these proteins, SPARC has been well studied in various cancers. Increasing evidence has emphasized the importance of SPARC in regulating proliferation, cell-cycle progression, apoptosis, adhesion, and cell-matrix interactions [9]. More interestingly, a number of studies have demonstrated that SPOCK1 plays a critical role in prostate cancer recurrence, glioblastoma invasion, and hepatocellular carcinoma progression [10-12]. However, the underlying mechanism of SPOCK1 overexpression is far from clear. Even less is known about the function and mechanism by which SPOCK1 contributes to cancer development and progression.

Considering the structural similarity between SPOCK1 and SPARC, it is of great interest to investigate the role of SPOCK1 in GBC development and progression. In the present study, we demonstrated a significant correlation between high expression of SPOCK1 and poor prognoses of GBC patients, and its oncogene function was examined further *in vitro* and *in vivo*. With a focus on its anti-apoptotic and epithelial-mesenchymal transition (EMT) functions, we demonstrated that SPOCK1 acts as a potential oncogene, which in turn contributes to the initiation and progression of GBC.

## Methods

### Patients, specimens, and cell lines

This study was approved by the ethics committee of Xinhua Hospital, and all patients provided informed consent. Cancer tissue specimens were obtained from 64 GBC patients who underwent radical cholecystectomy without prior radiotherapy or chemotherapy between 2010 and 2013 at the Department of General Surgery, Xinhua Hospital, School of Medicine, Shanghai Jiao Tong University, China. In addition, 60 patients with cholelithiasis who underwent simple cholecystectomy were included as controls. All diagnoses of GBC, cholelithiasis, and lymph node metastasis were confirmed by histopathological examination. The tissue specimens had been fixed in 4% formalin immediately after removal and embedded in paraffin for immunohistochemical staining. Fresh GBC tissue samples and paired non-cancerous tissue samples were obtained from 28 GBC patients. These samples were used for quantitative real-time PCR analysis (qRT-PCR). Fresh tissues were processed within 15 min after removal. Each sample was frozen and stored at −80°C. Paired non-cancerous tissues were dissected at least 2 cm away from the tumor border and were confirmed to lack tumor cells by microscopy. Among the 64 GC cases, there were 22 males and 42 females with ages ranging from 44 to 90 years (mean age: 68 years). All specimens and fresh tissue samples had been confirmed by pathological diagnosis and were staged according to the 7^th^ AJCC-TNM Classification of Malignant Tumors. The median follow-up period was 15 months (range, 1–36.5 months).

GBC cell lines GBC-SD, NOZ, SGC-996, OCUG, and EH-GB-1 were obtained from the Health Science Research Resources Bank (Osaka, Japan).

### Immunohistochemical analysis and evaluation of SPOCK1 expression

Immunohistochemical staining was performed using a standard immunoperoxidase staining procedure. SPOCK1 expression in benign and malignant specimens was evaluated according to methods described by Pinheiro et al. [13]. Sections were semi-quantitatively scored for the extent of immunoreactions as follows: 0, 0% immunoreactive cells; 1, <5% immunoreactive cells; 2, 5–50% immunoreactive cells; and 3, >50% immunoreactive cells. Additionally, the staining intensity was semi-quantitatively scored as 0 (negative), 1 (weak), 2 (intermediate), or 3 (strong). The final immunoreaction score was defined as the sum of both parameters, and the samples were grouped as negative (0), weak (1–2), moderate (3), and strong (4–6) staining. For statistical purposes, only the final immunoreaction scores of moderate and strong groups were considered as positive, and the other final scores were considered as negative.

### Quantitative real-time PCR

Total RNA was extracted from tissue samples or cultured cells with Trizol reagent (Takara, Shiga, Japan). cDNA was synthesized from 2 μg of total RNA using random primers and M-MLV Reverse Transcriptase (Invitrogen, Carlsbad, CA). RNA expression was measured by qRT-PCR using the SYBR-Green method (Takara) according to the manufacturer’s instructions. The relative expression level of the target gene was calculated by 2^-ΔCT^ (ΔC_T_ = C_T_^target^-C_T_^GADPH^) and normalized to the relative expression detected in the corresponding control cells, which was defined as 1.0. For the correlation study, the expression level (defined as the fold change) of SPOCK1 was calculated by 2^-ΔΔCT^ (ΔΔCT = ΔCT^tumor^-ΔCT^nontumor^). Primer sequences are listed in Additional file 1: Table S1.

### Lentivirus-mediated RNA interference

The short hairpin RNAs (shRNAs) [14] which listed in Additional file 1: Table S1 were used to target SPOCK1. The sequence of the negative control shRNA was 5′-TTCTCCGAACGTGTCACGT-3′. shSPOCK1-1 and negative control shRNA were synthesized and inserted into the pFH1UGW lentivirus core vector containing a cytomegalovirus-driven enhanced green fluorescent protein (EGFP) reporter gene. Expression of the shRNA was driven by the H1 promoter. Recombinant lentiviruses expressing SPOCK1-shRNA or negative control shRNA (Lv-shSPOCK1 and Lv-shNC, respectively) were produced by Genechem (Shanghai, China). GBC-SD and NOZ cells were infected with concentrated virus in serum-free medium. The supernatant was replaced with complete culture medium after 24 h. SPOCK1 expression in the infected cells was validated by qRT-PCR analysis and western blot assays after 120 h.

### Construction of plasmids and transfection

The full-length SPOCK1 cDNA (nt 152–1471; GenBank accession number NM_004598) was cloned into the GV143 expression vector (Genechem, Shanghai, China) and transfected into SGC-996 cells. Stable SPOCK1-expressing clones were selected for 2 weeks using neomycin (Genechem), and the expression level of SPOCK1 was determined by qRT-PCR and western blot assays. Empty vector-transfected cells (MOCK) were used as control. Primer sequences for vectors construction are listed in Additional file 1: Table S1.

### In vitro tumorigenesis assays

A Cell Counting Kit-8 (CCK-8; Dojindo) cell proliferation assay was performed according to the manufacturer’s instructions. Anchorage-independent growth was assessed by a colony formation assay. Briefly, 500 cells were seeded in 6-well plates. The cells were cultured for approximately 14 days, fixed with 4% paraformaldehyde, and stained with 0.1% crystal violet (Sigma, St. Louis, MO). The total number of colonies (>50 cells/colony) was counted. Edu retention assays were performed to examine the effect of SPOCK1 on DNA replication. Dissociated cells were exposed to 25 μM of 5-ethynyl-2′-deoxyuridine (Edu, RiboBio, Guangzhou, China) for 2 hr at 37°C, and then the cells were fixed in 4% paraformaldehyde. After permeabilization with 0.5% Triton-X, the cells were reacted with 1× Apollo reaction cocktail (RiboBio) for 30 min. Subsequently, the DNA contents of the cells were stained with Hoechst 33342 for 30 min and visualized under a fluorescence microscope. The experiments were performed in triplicate.

### In vitro migration and invasion assays

For the *in vitro* wound-healing assay, a cell-free area of the culture medium was wounded by scratching with a 200-μL pipette tip. Cell migration into the wound area was monitored in serum-free medium and photographed under a fluorescence microscope at 0 and 48 h. Cell migration and invasion were examined using 8-μm transwell filters (BD Biosciences, Franklin Lakes, NJ). GBC-SD (3 × 10^4^), NOZ (4 × 10^4^) cells, and SGC-996 (8 × 10^4^) in 0.5 μL serum-free medium were added to the upper chamber containing an uncoated or Matrigel (BD Biosciences)-coated membrane. The lower chamber was filled with 500 μL basal medium with 10% fetal bovine serum (FBS). After 24 h of incubation at 37°C in a humidified 5% CO_2_ incubator, cells that migrated to the lower compartment were fixed with methanol and stained with crystal violet. Migrated or invaded cells were counted in five randomly chosen fields in each well. Imaging and cell counting were performed at × 10 magnification under a fluorescence microscope. The experiments were performed in triplicate.

### Subcutaneous and peritoneal xenograft models

Nude nu/nu mice, 4–6 weeks old, were purchased from the Shanghai Laboratory Animal Center of the Chinese Academy of Sciences (Shanghai, China). All mice were housed in specific pathogen-free conditions following the guidelines of the Ethics Committee of Xinhua Hospital, School of Medicine, Shanghai Jiaotong University. To explore the effects of SPOCK1 on tumor growth *in vivo*, cells were subcutaneously injected into the left axilla of the mice (five mice/group). Tumor growth was monitored every week and measured in two dimensions. The tumor volume was calculated using the following formula: tumor volume = 4π/3 × (width/2)^2^ × (length/2), where the width and length were the shortest and longest diameters, respectively. After 4 weeks, the mice were sacrificed and the tumors were dissected out and weighed. The proliferative index of Ki-67 was evaluated in xenograft tumors by immunohistochemical staining (IHC). In addition to investigating the effects of SPOCK1 on tumor metastasis *in vivo*, 1 × 10^5^ NOZ cells (Lv-shNC and Lv-shSPOCK1) were suspended in 1 mL serum-free medium and peritoneally injected into five mice. After 4 weeks, the mice were sacrificed and the peritoneal tumors were collected for IHC.

### Immunofluorescence analysis

Cells were seeded in 6-well plates and cultured overnight. Then, the cells were fixed in 3.5% paraformaldehyde and permeabilized in a solution of 0.1% bovine serum albumin (BSA) and 0.5% Triton X-100 at room temperature. After the blocking solution was washed out, the cells were incubated with primary antibodies against SPOCK1, E-cadherin, or vimentin for 60 min at 37°C and then washed with 0.1% BSA twice. After 60 min of incubation at 37°C with Cy3 Goat Anti-Rabbit IgG (Beyotime, Shanghai, China) and then washing with 0.1% BSA, the cells were counterstained with DAPI and imaged under a fluorescence microscope. The experiments were performed in triplicate.

### Flow cytometric analysis of cell apoptosis

The extent of apoptosis was measured with an Annexin V-APC Apoptosis Detection kit (BD Biosciences) according to the manufacturer′s instructions. Untransfected and transfected GBC-SD, SGC-996, and NOZ cells were collected, washed with cold PBS twice, gently resuspended in 100 μL of 1× binding buffer containing 2.5 μL APC-conjugated annexin-V and 1 μL of 100 μg/mL PI, and then incubated at room temperature in the dark for 15 min. The stained cells were analyzed by flow cytometry (BD Biosciences). The experiments were performed in triplicate.

### Nuclear morphology assay

Untransfected and transfected GBC-SD and NOZ cells were seeded in 6-well culture plates. After 48 h, the cells were washed with PBS, fixed in MeOH-HOAc (3:1, v/v) for 10 min at 4°C, and stained with Hoechst 33342 (5 μg/mL in PBS) for 5 min at room temperature. The stained cells were then examined under a fluorescence microscope. The experiments were performed in triplicate.

### Antibodies and western blotting

A rabbit anti-SPOCK1 antibody was purchased from Abcam (MA, USA). Rabbit anti-Snail, anti-vimentin, anti-N-cadherin, anti-E-cadherin, anti-PI3K, anti-phospho-PI3K (Tyr607), anti-Akt, anti-phospho-Akt (Ser473), anti-Bax, anti-Bcl-2, anti-cleaved caspase 3 and 9, anti-poly (adenosine diphosphate-ribose) polymerase (PARP), and anti-GADPH antibodies were obtained from Cell Signaling Technology (Danvers, USA). Briefly, equal quantities of cellular proteins were resolved by sodium dodecyl sulfate-polyacrylamide gel electrophoresis, transferred onto polyvinylidene difluoride membranes, and immunoblotted with a primary antibody. After incubation with a secondary antibody, blots were visualized by enhanced chemiluminescence (Millipore, Billerica, MA). GADPH was used as the loading control.

### Statistical analysis

All statistical analyses were performed using SPSS 19.0 software. SPOCK1 mRNA levels in tumor and paired nontumor tissues were compared with the paired Student’s t-test. The independent Student’s t-test was used to compare the means of two groups. The Pearson χ^2^ test was used to analyze the association of SPOCK1 expression with clinicopathologic parameters. Kaplan-Meier plots and log-rank tests were used for survival analysis. Univariate and multivariate Cox proportional hazard regression models were used to analyze independent prognostic factors. Each experimental value was expressed as the mean ± standard deviation (SD). Differences between groups were considered significant at *P* < 0.05. All data points represent the mean of triplicate data points.

## Results

### Clinical significance of SPOCK1 in GBC

**Figure 1:**
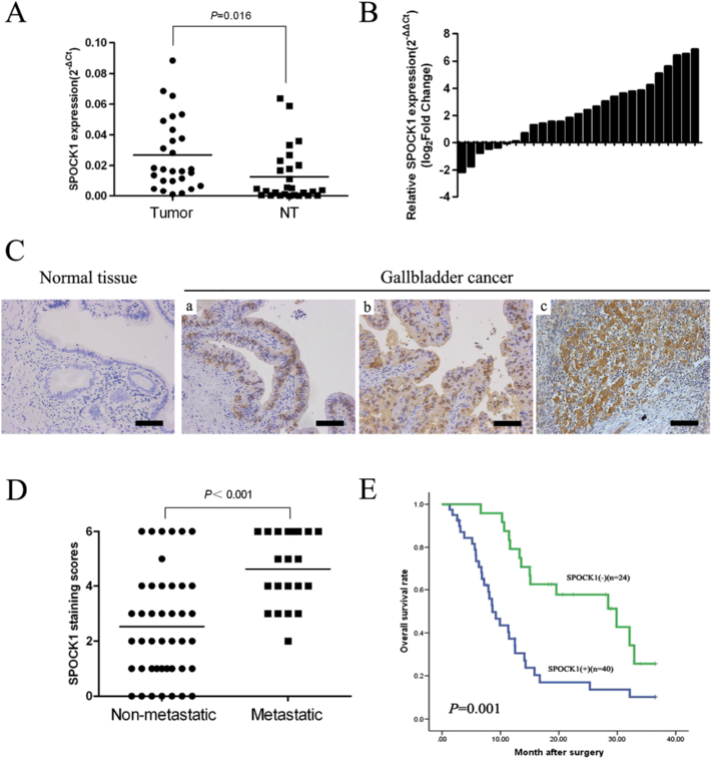
**Clinical significance of SPOCK1 in human GBC. (A)** Scatterplots of the relative expression of SPOCK1 in GBC tissues and their matched nontumor counterparts. SPOCK1 expression was calculated and is expressed as the SPOCK1/GADPH expression ratio (2^-ΔCT^). **(B)** Comparison of the SPOCK1 expression level between GBC tissues and their corresponding nontumor tissues. **(C)** Representative image of GBC staining with an anti-SPOCK1 antibody. (a, b) Weak expression of SPOCK1 in well and moderately differentiated GBC tissues; (c) strong expression of SPOCK1 in poorly differentiated GBC tissues (scale bar, 100 μm). **(D)** Scatterplots of the average staining scores of SPOCK1 expression in patients with or without metastasis. **(E)** Kaplan-Meier overall survival curve of GBC patients correlated with SPOCK1 expression.

To assess the role of SPOCK1 in GBC, SPOCK1 mRNA expression in 28 pairs of GBCs (tumor and corresponding nontumor tissues) was compared by qRT-PCR. The relative expression level of SPOCK1 was significantly higher in tumor tissues compared with that in their nontumor counterparts (*P* = 0.016) (Figure 1A and B). Furthermore, protein expression levels of SPOCK1 were measured in 64 samples of archived paraffin-embedded GBC tissues and 60 cholecystitis gallbladder epithelial tissues by immunohistochemistry (Figure 1C). SPOCK1 expression was significantly higher in tumor tissues compared with that in cholelithiasis tissues (*P* = 0.002) (Table 1). A clinicopathological association study of the 64 GBCs found that SPOCK1 was significantly associated with histological differentiation (*P* = 0.012) (Figure 1C) and lymph node metastasis (*P* < 0.001) (Table 2). GBC patients who developed metastasis showed significantly higher immunoreaction scores for SPOCK1 than those without metastasis (*P* < 0.001) (Figure 1D), which implies that SPOCK1 might play a role in metastasis. More intriguingly, positivity for SPOCK1 was correlated significantly with shorter overall survival (OS) (log rank, 11.301; *P* = 0.001) (Figure 1E). Multivariate Cox regression analysis further revealed that SPOCK1 was an independent prognostic marker for the OS time of GBC patients (hazard ratio, 0.378; 95% confidence interval, 0.164–0.871; *P* = 0.022) (Table 3).

**Table 1:** **Immunohistochemical analysis of SPOCK1 expression in GBC**

**Group**	**No.of cases**	**SPOCK1 expression**				***p***
		**Negative (0)**	**Weakly stained (1–2)**	**Moderately stained (3)**	**Strongly stained (4–6)**	
Cholecystitis tissues	60	48	12	0	0	0.002
Gallbladder cancer	64	7	17	11	29

**Table 2:** **Association of SPOCK1 expression with the clinicopathological characteristics of GBC**

**Variable**	**Category**	**No. of cases**	**SPOCK1**		
			**No. of positive cases (%)**	**χ2**	***p***
Age	<60	18	11 (61.1)	0.021	0.886
	≥60	46	29 (59.2)		
Sex	Male	22	14 (63.6)	0.018	0.892
	Female	42	26 (61.9)		
Jaundice	Present	33	21 (63.6)	0.038	0.846
	Absent	31	19 (61.3)		
Associated gallstone	Present	42	25 (59.5)	0.462	0.497
	Absent	22	15 (68.2)		
**Histology differentiation**	**Well or moderate**	**43**	**22 (51.2)**	**7.187**	**0.012**
	**Poor**	**21**	**18 (85.7)**		
Tumor invasion(AJCC)	Tis-T_1_	15	7 (46.7)	2.096	0.148
	T_2_-T_4_	49	33 (67.3)		
**Lymph node metastasis**	**Absent**	**43**	**20 (46.5)**	**14.293**	**<0.001**
	**Present**	**21**	**20 (95.2)**		
TNM stage(AJCC)	0-I	15	7 (46.7)	2.096	0.148
	II-IV	49	33 (67.3)		
Total	64	40 (62.5)			

Bold values indicate statistical significance, *P* < 0.05.

**Table 3:** **Univariate and multivariate analysis of the association of prognosis with clinicopathologic parameters and SPOCK1 expression in GBC patients**

**Variable**	**Univariable analysis**		**Multivariable analysis**	
	**HR (95% CI)**	***p***	**HR (95% CI)**	***p***
Age (<60 *vs.* ≥60)	0.502 (0.240-1.051)	0.062	**-**	**-**
Sex (male *vs.* female)	1.076 (0.575-2.017)	0.818	-	-
Jaundice (present *vs.* absent)	1.324 (0.780-2.409)	0.356	-	-
Associated gallstone (present *vs.* absent)	0.550 (0.294-1.030)	0.058	-	-
**Histology differentiation (well or moderate** ***vs. *** **poor)**	**0.431 (0.233-0.796)**	**0.006**	1.480 (0.721-3.038)	0.286
**Tumor invasion (AJCC) (Tis-T1** ***vs.*** **T2-T4)**	**6.272 (2.507-15.691)**	**<0.001**	**8.136 (2.584-25.619)**	**<0.001**
**Lymph node metastasis (present** ***vs.*** **absent)**	**6.278 (3.126-12.611)**	**<0.001**	**2.653 (1.100-6.396)**	**0.030**
**TNM stage (AJCC) (0-I** ***vs.*** **II-IV)**	**6.272 (2.507-15.691)**	**<0.001**	**8.136 (2.584-25.619)**	**<0.001**
Type of surgery (curative resection *vs.* palliative)	0.687 (0.361-1.307)	0.249	-	-
**Overexpression of SPOCK1 in tumor**				
**(Negative** ***vs.*** **positive)**	**0.346 (0.181-0.659)**	**0.001**	**0.378 (0.164-0.871)**	**0.022**

Bold values indicate statistical significance, *P* < 0.05, CI, confidence interval; HR, hazard ratio.

### SPOCK1 expression in GBC cell lines

**Figure 2:**
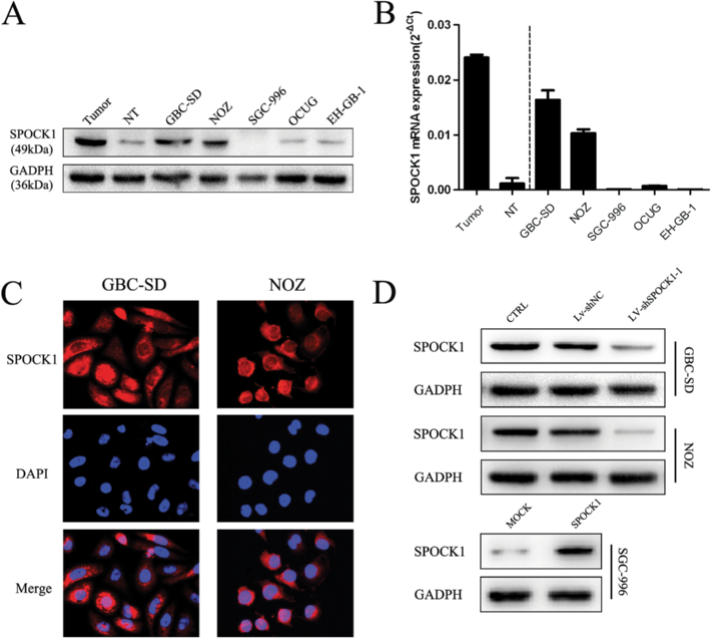
**Expression of SPOCK1 in GBC cell lines. (A and B)** mRNA and protein expression of SPOCK1 in tumor and nontumor tissues, and GBC cell lines GBC-SD, NOZ, SGC-996, OCUG, and EH-GB-1. **(C)** Immunofluorescence images of SPOCK1 (red) and DAPI (blue) staining in GBC-SD and NOZ cells (×200). **(D)** Western blot analysis of SPOCK1 expression in SPOCK1-depleting GBC-SD and NOZ cells and SPOCK1 overexpressing SGC-996 cells. GADPH was used as the loading control.

To explore the role of SPOCK1 in the progression of GBC, we detected the endogenous expression of SPOCK1 in tumor and nontumor tissues, and five GBC cell lines by RT-PCR and western blotting. Among five GBC cell lines, SPOCK1 expression was overexpressed in GBC-SD and NOZ cells and gradually decreased in OCUG, EH-GB-1, and SGC-996 (Figure 2A). Consistent protein levels were observed in western blotting (Figure 2B). In addition, SPOCK1 protein immunostaining was mainly found on the cell membrane and in the cytoplasm of GBC cells (Figure 2C). We chose GBC-SD and NOZ cell lines for stable transfection with shRNA lentivirus vectors toward SPOCK1, and SGC-996 cell lines for stable transfection with SPOCK1-expression vector. The effect of shRNA transduction on the expression of SPOCK1 was examined using RT-PCR analysis with the most efficient knockdowns by shSPOCK1-1 in GBC-SD and NOZ cell lines compared with those of the other two vectors (Additional file 2: Figure S1B). We examined the lentiviral transduction efficiency by EGFP expression under a microscope at 72 h after transduction (Additional file 2: Figure S1A). The efficiency of lentiviral transduction in both GBC-SD and NOZ cell lines was higher than 90%. The transfection efficiency was comfirmed by RT-PCR (Additional file 2: Figure S1B) and western blotting (Figure 2D).

### Effects of SPOCK1 overexpression and knockdown on GBC cell growth in vitro and in vivo

**Figure 3:**
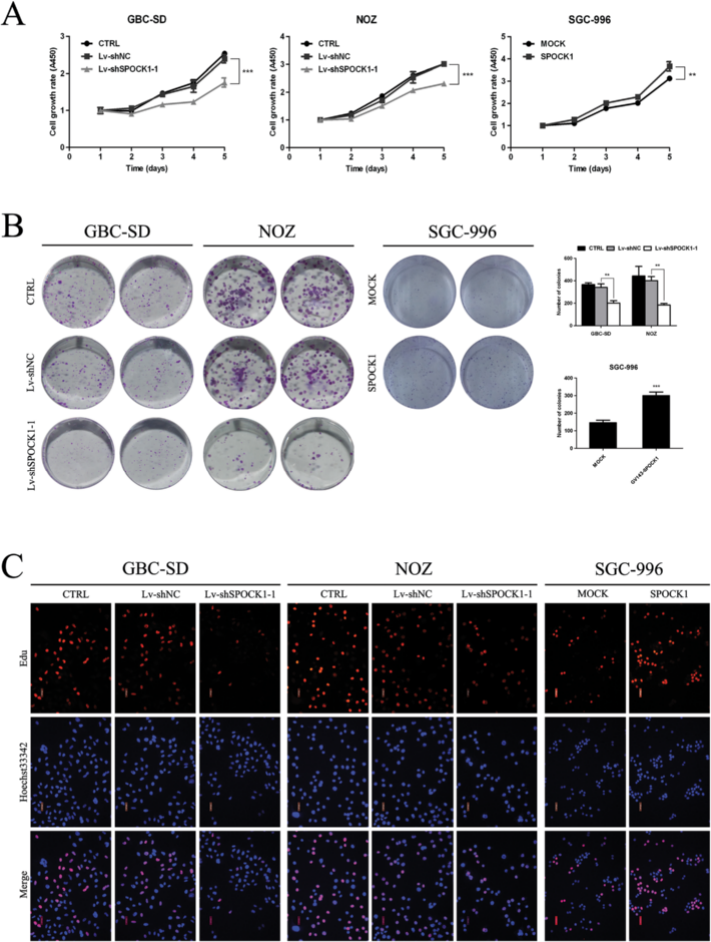
**Effect of SPOCK1 overexpression and silencing on the growth of GBC cells** ***in vitro*** **. (A)** The cells growth rates were determined by CCK-8 proliferation assays at various time points (*P < 0.05, **P < 0.01, and ***P < 0.001). **(B)** Representative images of colony formation induced by Lv-shNC, Lv-shSPOCK1, MOCK-SGC-996, and SPOCK1-SGC-996 cells. The numbers of colonies were calculated and are depicted in the bar chart (*P < 0.05, **P < 0.01, and ***P < 0.001). **(C)** Knockdown of SPOCK1 expression inhibited DNA replication in GBC-SD and NOZ cells compared to control as determined by the EdU incorporation assay. Elevated expression of SPOCK1 increased DNA replication in SGC-996 cells.

**Figure 4:**
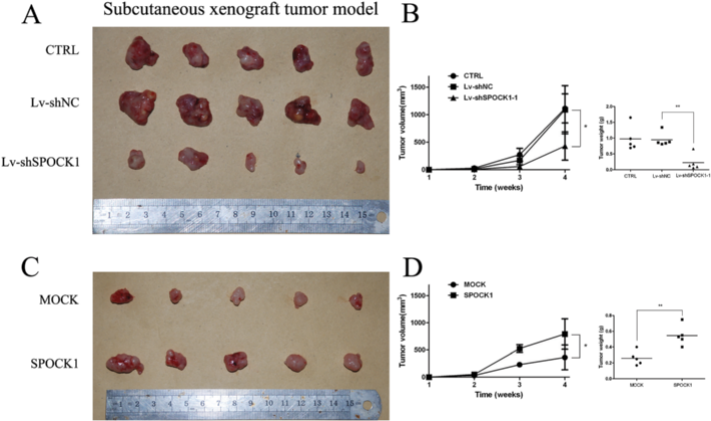
**Effect of SPOCK1 overexpression and silencing on the growth of GBC cells** ***in vivo*** **. (A and C)** Representative examples of tumors formed in nude mice injected with the indicated cells. **(B and D)** Tumor growth curves are summarized in the line chart. A statistical plot of average tumor weights in the subcutaneous xenograft model (*P < 0.05, **P < 0.01, and ***P < 0.001).

The tumorigenic ability of SPOCK1 was assessed by CCK-8 and colony formation assays. Figure 3A shows that the proliferation of GBC-SD and NOZ cells was significantly inhibited by SPOCK1 depletion (P < 0.001). Additionally, compared with CTRL and Lv-shNC groups, the colony formation assay showed that the number of colonies formed by GBC-SD and NOZ cells was significantly decreased by silencing SPOCK1 (Figure 3B) (P < 0.01). To investigate the mechanisms underlying altered cell growth, EdU retention assays were performed to examine the regulatory effect of SPOCK1 on DNA replication. Following transfection with Lv-shSPOCK1, the percentage of EdU-positive cells was reduced in GBC-SD and NOZ cells compared to the controls (Figure 3C). Furthermore, to assess the effects of SPOCK1 on GBC growth *in vivo*, SPOCK1-depleted or control NOZ cells were injected into the left axilla of nude mice, and then the tumor volume was monitored. Our results showed that the growth of SPOCK1-depleted xenografts was significantly inhibited compared with that of tumors formed by control cells (Figure 4A and B). Moreover, IHC staining showed less Ki-67 positive cells in SPOCK1-depleted inoculated tumor tissues (Additional file 3: Figure S2B). Conversely, compared with empty vector-transfected cells, SPOCK1-transfected cells showed increased growth rates (Figure 3A) (P < 0.01), greater colony forming abilities (Figure 3B) (P < 0.001), increased DNA replication (Figure 3C), larger mean tumor volume (Figure 4B) (P < 0.05) and higher Ki-67 index (Additional file 3: Figure S2B). Collectively, our data demonstrate that SPOCK1 plays a potential role in promoting cell proliferation both *in vitro* and *in vivo*.

### SPOCK1 promotes cell migration and invasion in vitro and in vivo by inducing EMT

**Figure 5:**
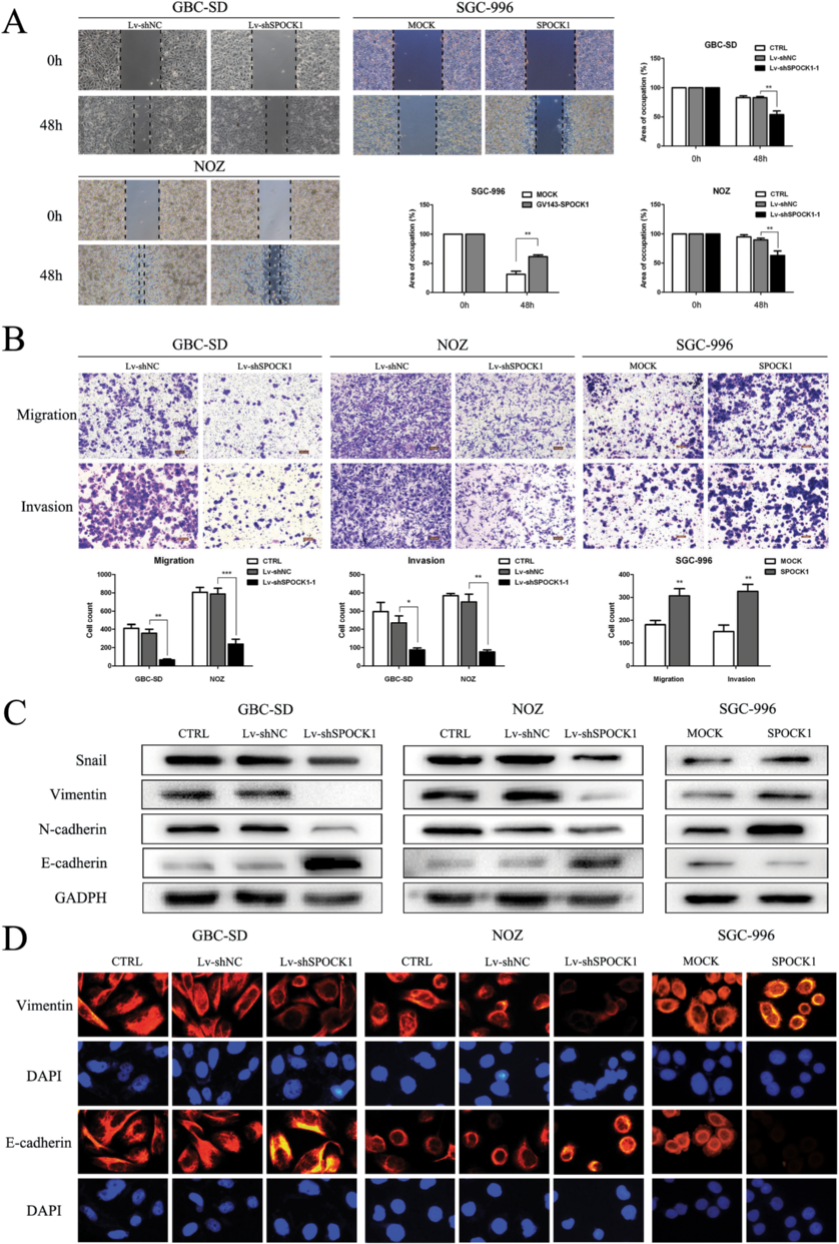
**SPOCK1 promotes tumor invasion and metastasis** ***in vitro*** **by inducing EMT. (A)** Wound closure was delayed in Lv-shSPOCK1-transduced cells compared with that in CTRL and Lv-shNC cells at 48 h in both GBC-SD and NOZ cells. Overexpression of SPOCK1 in SGC-996 cells had the opposite effects (*P < 0.05, **P < 0.01, and ***P < 0.001). **(B)** Matrigel invasion assay of CTRL, Lv-shNC, Lv-shSPOCK1, MOCK, and SPOCK1 transfectants cells. The number of invaded cells was calculated and is depicted in the bar chart. (*P < 0.05, **P < 0.01, and ***P < 0.001). **(C and D)** The protein expression of Snail, vimentin, N-cadherin and E-cadherin in the indicated cells was examined by western blotting. The protein expression of vementin and E-cadherin was examined by immunofluorescence analysis. The red signal represents staining for E-cadherin or vimentin. Nuclei were counterstained with DAPI.

**Figure 6:**
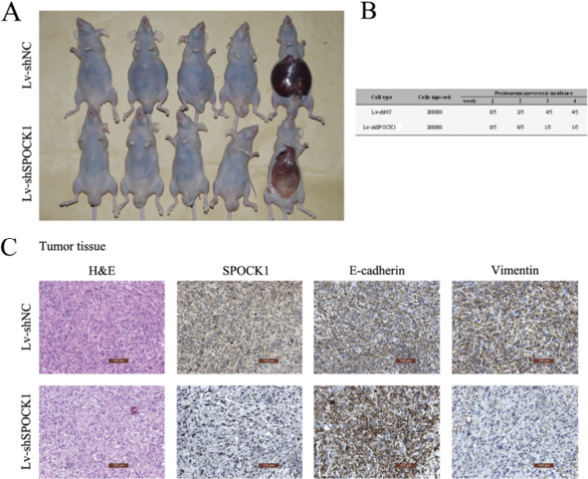
**SPOCK1 promotes tumor invasion and metastasis** ***in vivo*** **by inducing EMT. (A)** An *in vivo* metastasis assay was performed to evaluate the effect of Lv-shSPOCK1 cells on tumor metastasis. Mice that received SPOCK1-depleted NOZ cells exhibited little ascites at 4 weeks after implantation. **(B)** The tumor incidence rate during the 4-week observation period. **(C)** Immunohistochemical staining of SPOCK1, E-cadherin, and vimentin in tumor tissues of the peritoneal metastasis model.

To investigate the effects of SPOCK1 on cancer cell migration and invasion, we performed *in vitro* wound healing and transwell migration assays, and an *in vivo* metastasis assay. Both wound healing and transwell migration assays showed that the invasive capability of control cells was greater than that of the transfected cells, while overexpression of SPOCK1 in SGC-996 cells showed the opposite effect (Figure 5A and B). These results indicate that SPOCK1 increases cell invasion. To determine whether SPOCK1 promoted the invasiveness of GBC through EMT processes, we detected EMT biomarkers by immunofluorescence analysis and western blotting. Consistently, we found that both GBC-SD and NOZ cells transfected with shSPOCK1 expressed high levels of E-cadherin, which is characteristic of epithelial cells. However, in GBC cell lines transfected with shSPOCK1, there was a decrease in the expression of Snail, Vimentin and N-cadherin, indicating a mesenchymal phenotype (Figure 5C and D). Overexpression of SPOCK1 could reverse this phenotype (Figure 5C and D). To confirm these findings *in vivo*, we used a peritoneal metastasis model in nude mice. Mice injected with SPOCK1-depleted NOZ cells exhibited few ascites (Figure 6A) at 4 weeks after implantation. After dissecting out the peritoneal metastatic tumors, the tumors were analyzed by histology (Figure 6C). In the immunohistochemical analysis, the Lv-shSPOCK1 group showed very light staining of SPOCK1 and vimentin (Figure 6C), which was consistent with the results of western blotting and immunofluorescence analysis *in vitro*. Our data suggest that SPOCK1 promotes tumor invasion and metastasis via inducing EMT.

### SPOCK1 inhibits apoptosis in GBC cells

**Figure 7:**
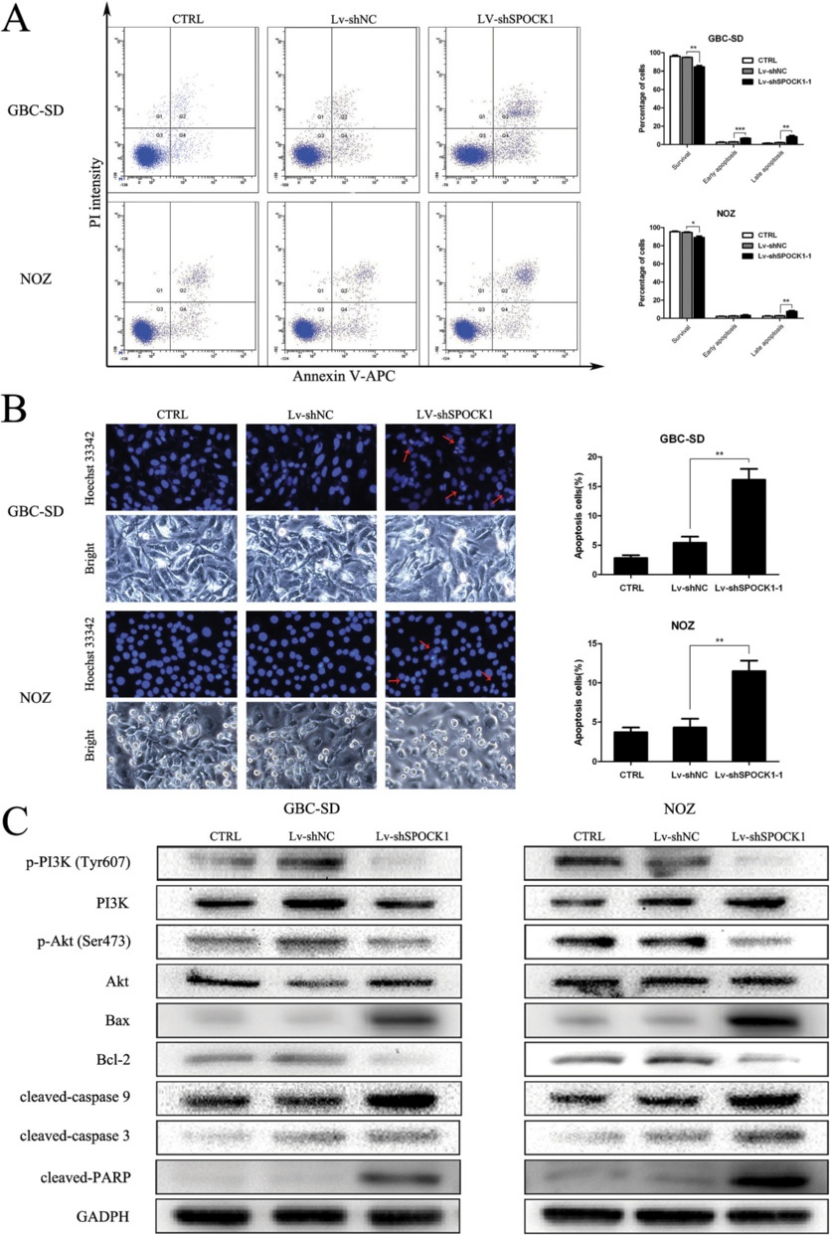
**SPOCK1 exerts an anti-apoptotic effect via the PI3K/Akt pathway. (A)** Apoptosis was determined by flow cytometry. Cells stained with annexin-V-APC were considered as apoptotic. The apoptotic index was defined as the percentage of apoptotic cells. **(B)** Apoptotic changes in the nuclear morphology of GBC-SD and NOZ cells as indicated by Hoechst 44322 staining (blue). The apoptotic index, defined as the percentage of apoptotic cells, was calculated and is summarized in the bar chart (*P < 0.05, **P < 0.01, and ***P < 0.001). **(C)** The levels of phosphorylated PI3K (Tyr607), total PI3K, phosphorylated Akt (Ser473), total Akt, Bax, Bcl-2, caspase-9, caspase-3, and PARP were detected in CTRL, Lv-shNC, and Lv-shSPOCK1 cells by western blot analysis. GADPH was used as the loading control.

To explore the molecular mechanism by which SPOCK1 regulated the proliferation and metastasis of GBC cells, we investigated the effect of SPOCK1 on apoptosis. The apoptotic indexes of knockdown control cells (Lv-shNC) and SPOCK1-silenced cells (Lv-shSPOCK1) were 4.86% and 15.43% (GBC-SD, P < 0.01), 5.3% and 10.77% (NOZ, P < 0.05), respectively (Figure 7A). Furthermore, the apoptotic index of SPOCK1 transfectants in SGC-996 cells was lower than that of vector transfectants (Additional file 4: Figure S3A). These results indicate that silencing SPOCK1 restores the cellular response to apoptotic stimuli. Phase contrast microscopic observation of SPOCK1-silenced cells showed that the growth inhibitory effect was accompanied by cell shrinkage (Figure 7B), suggesting apoptotic cell death. Control and negative control cells were normal with round and homogeneous nuclei, whereas SPOCK1-silenced cells exhibited the hallmark characteristics of apoptosis with cell shrinkage, and nuclear condensation and fragmentation (Figure 7B).

### SPOCK1 exerts an anti-apoptotic effect through the PI3K/Akt pathway

Tumor cells resist cell death through either disruption of apoptotic processes or activation of survival signals. In general, survival signals are mediated by the PI3K/Akt pathway [15]. Deregulation of Akt phosphorylation represents an important anti-apoptotic mechanism in various cancers. Activated Akt can phosphorylate a wide variety of substrate proteins including Bax, a pro-apoptotic member of the Bcl-2 protein family, which is suppressed by phosphorylation. Bax inactivation maintains the integrity of the mitochondrial membrane, which activates caspase-9, caspase-3, and PARP [16]. Therefore, we examined whether SPOCK1 inhibits apoptosis via PI3K and Akt phosphorylation. Compared with control cells, the levels of both phosphorylated PI3K (Tyr607) and Akt (Ser473) were decreased in SPOCK1-transfected cells, while their total protein levels were unaffected. Inactivated Akt subsequently regulates Bcl-2 family proteins. As a result, the subsequent cleavages of caspase-9, caspase-3, and PARP were all increased in SPOCK1 knockdown cells compared with those in control cells (Figure 7C). The PI3K and Akt phosphorylation were reversed when SPOCK1 was overexpressed in SGC-996 cells (Additional file 4: Figure S3B). These results indicated that the PI3K/Akt pathway might participate in the SPOCK1-induced anti-apoptotic effect on GBC cells.

## Discussion

GBC is a highly lethal disease, and most afflicted individuals do not survive because of local tumor spread and invasion. Therefore, efforts are urgently needed to identify reliable tumor markers for early detection and cancer-specific cellular targets for novel therapeutic approaches. SPOCK1 is a proteoglycan that was first isolated in human testes and initially called ‘tesyican’. It is dysregulated in many organs and tissues including the brain, cartilage, vascular endothelium, lymphocytes, and neuromuscular junctions [11,12,17]. Recently, SPOCK1 was also found to be overexpressed in hepatocellular carcinomas [18]. Although SPOCK1 is reported to be overexpressed in several other types of carcinoma, it has not been linked to GBC or any other malignancy of the biliary tract.

Our clinical association study found that SPOCK1 was highly expressed in GBC tissues compared with that in their nontumor counterparts, indicating that SPOCK1 might play a role in GBC development. Moreover, immunohistochemistry showed that overexpression of SPOCK1 was significantly associated with histological differentiation, lymph node metastasis, and a shorter OS time of GBC patients. Cox proportional hazard regression analysis further identified SPOCK1 as an independent factor for poor prognosis. Because SPOCK1 is a secreted protein that can be detected at very low levels in normal tissues, SPOCK1 overexpression in GBC may serve as a biomarker for early detection and precise prognoses.

In this study, we confirmed that SPOCK1 was expressed in GBC by qRT-PCR, western blotting, and immunofluorescence, which represented an ideal model to study the role and molecular mechanisms of SPOCK1. A series of *in vitro* and *in vivo* assays showed that cancer cell growth, DNA replication and the colony formation capability were significantly decreased by inhibition of SPOCK1, suggesting its role in cancer cell proliferation and tumor growth. Additionally, we found that SPOCK1 induced GBC cells migration and invasion, indicating that SPOCK1 might undergo metastasis-related genetic alteration in GBC cells. Metastasis is a multistep cellular process and the most common cause of death in GBC patients. This process involves the spread of tumor cells from a primary tumor to a secondary site within the body. It usually involves a variety of complicated molecular and cellular factors related to cell proliferation and migration, degradation of the basement membrane, invasion, adhesion and angiogenesis. At the molecular level, the acquisition of genetic and/or epigenetic alterations, along with the cooperation of stromal cells, contribute to cancer metastasis [19,20].

SPOCK1 promotes cancer cell migration by induction of EMT [21]. EMT is a crucial step in tumor progression and plays a critical role during cancer invasion and metastasis. During this process, epithelial cells lose their properties and acquire mesenchymal phenotypes. Mesenchymal phenotype cells exhibit increased expression of mesenchymal-related markers, such as vimentin, and decreased expression of epithelial-related markers such as E-cadherin [22,23]. In the current study, we showed that suppressed expression of SPOCK1 induced EMT by elevating expression of the epithelial marker E-cadherin and reducing expression of the mesenchymal marker Snail, vimentin and N-cadherin. Our findings indicate that SPOCK1 may drive EMT in cancer cells, resulting in metastasis.

Further experiments revealed that SPOCK1-enhanced tumor cell survival may be attributable to its anti-apoptotic effect. Our data show that SPOCK1 contributes to anti-apoptotic effects through inactivation of the PI3K/Akt pathway, which subsequently activates the caspase 9/caspase 3/PARP pathway. Inhibition of apoptosis is one of the major mechanisms in cancer development and ultimately leads to the expansion of neoplastic cells with deregulated proliferation and accumulation of genetic instability and mutations [24]. Therefore, impaired GBC cell growth and metastasis due to SPOCK1 knockdown can be explained, at least in part, by inactivation of the PI3K/Akt pathway. Previous reports show that PI3K/Akt is a classical signaling pathway [25-27], and its activation induces cell growth [28,29], promotes EMT [30], and stimulates Bax-mediated signaling for apoptosis progression. Our results suggest that inactivation of PI3K/Akt signaling is responsible for SPOCK1 shRNA-mediated suppression of tumor cell proliferation, migration, invasion, and EMT.

Because SPOCK1 belongs to the Ca^2+^-binding proteoglycan family, some of these effects may be mediated by the glycan segment of SPOCK1. Increasing evidence has shown that glycan specifically interacts with growth factors, chemokines, and the matrix architecture [31]. Cancer cells usurp these properties to gain a survival advantage and invade tissues throughout the organism. For example, the glycan segment of perlecan protects fibroblast growth factor 2 from proteolytic degradation and potentiates its angiogenic role [32]. In addition to the steady-state properties of glycan, changes in glycan segments affect cancer development, such as glycosylation and depolymerization. Heparanase-induced depolymerization can release fibroblast growth factor 2 from perlecan to facilitate vascular sprouting during angiogenesis [33]. Some of these characteristics of perlecan may be shared by other pericellular proteoglycans such as agrin, collagen type XVIII, and SPOCK1. However, to determine whether SPOCK1 performs its functions by working alone or in concert with other partner molecules, it will be important to identify the portion of the proteoglycan that mediates the interaction.

A better understanding of the oncogenic mechanisms of SPOCK1 during GBC initiation and progression may have implications for future patient treatments.

## Conclusions

We have demonstrated that the expression of SPOCK1 is associated with histological differentiation, lymph node metastasis, and the OS time of GBC patients. SPOCK1 promotes GBC cell proliferation and metastasis both *in vitro* and *in vivo*. We hypothesize that SPOCK1 might play an important role during the EMT of GBC cells, which results in metastasis. Moreover, SPOCK1 contributes to anti-apoptotic effects through inactivation of the PI3K/Akt pathway. These observations support our belief that SPOCK1 may serve as an oncogene in GBC pathogenesis.

## Additional files

Additional file 1: Table S1. The nucleotides applied in the study. Additional file 2: Figure S1. RT-PCR of the transfection efficiency in GBC cell lines. (A) The transduction efficiency was determined at 3 days after infection with lentiviruses at a multiplicity of infection of 50. The transduced cells labeled with EGFP were observed under a fluorescence microscope. Light micrograph (upper); fluorescent micrograph (lower) (×100). (B) RT-PCR were performed to detect SPOCK1 expression in the indicated cells (**P* < 0.05, ***P* < 0.01, and ****P* < 0.001). Additional file 3: Figure S2. Association of SPOCK1 expression on the growth of GBC cells *in vivo*. (A and B) Photograph of a subcutaneous xenograft model of human GBC in mice. (C) Immunohistochemical staining of Ki-67in tumor tissues of the subcutaneous xenograft model. Additional file 4: Figure S3. SPOCK1 exerts an anti-apoptotic effect via the PI3K/Akt pathway in SGC-996 cells. (A) Apoptosis was determined in empty vector- and SPOCK1-transfected cells by flow cytometry. The apoptotic index was defined as the percentage of apoptotic cells. (B) The levels of phosphorylated PI3K (Tyr607), total PI3K, phosphorylated Akt (Ser473), total Akt were detected in empty vector- and SPOCK1-transfected cells by western blot analysis. GADPH was used as the loading control.
