# Supplementary material for: SPOCK1 as a potential cancer prognostic marker promotes the proliferation and metastasis of gallbladder cancer cells by activating the PI3K/AKT pathway
Source: Mol Cancer. 2015 Jan 27;14(1):12. doi: 10.1186/s12943-014-0276-y (PMC4320842; doi:10.1186/s12943-014-0276-y)
Supplement: Additional file 1: Table S1. — The nucleotides applied in the study. [file 12943_2014_276_MOESM1_ESM.doc]

**Additional file 1: Table S1.The nucleotides applied in the study.**

| Description | Name | Sequence |
| --- | --- | --- |
| Primers for qRT-PCR | SPOCK1-F | 5'-CAACTGCTTGTTCCCAGAGG-3' |
|  | SPOCK1-R | 5'-GCCAATGACTTCCCTATCCA-3' |
|  | GADPH-F | 5'-AGAAGGCTGGGGCTCATTTG-3' |
|  | GADPH-R | 5'-AGGGGCCATCCACAGTCTTC-3' |
| shRNA for SPOCK1 | shRNA-1 | 5'-GUAAUGAGGAGGGCUAUUA-3' |
|  | shRNA-2 | 5'-GAUGCGAACAGAGUCAUCA-3' |
|  | shRNA-3 | 5'-GGACCUUCGAAUUUGGUCA-3' |
| Primers for vectors construction | GV143-SPOCK1-F | 5'-TACCGGACTCAGATCTCGAGATGCCGGCGATCGCGGTGTTG-3' |
|  | GV143-SPOCK1-R | 5'-GATCCCGGGCCCGCGGTACCGTCCATATGTACCCGACCTCATC-3' |
