# Supplementary material for: SPOCK1 as a potential cancer prognostic marker promotes the proliferation and metastasis of gallbladder cancer cells by activating the PI3K/AKT pathway
Source: Mol Cancer. 2015 Jan 27;14(1):12. doi: 10.1186/s12943-014-0276-y (PMC4320842; doi:10.1186/s12943-014-0276-y)
Supplement: Additional file 2: Figure S1. — RT-PCR of the transfection efficiency in GBC cell lines. (A) The transduction efficiency was determined at 3 days after infection with lentiviruses at a multiplicity of infection of 50. The transduced cells labeled with EGFP were observed under a fluorescence microscope. Light micrograph (upper); fluorescent micrograph (lower) (×100). (B) RT-PCR were performed to detect SPOCK1 expression in the indicated cells (*P < 0.05, **P < 0.01, and ***P < 0.001). [file 12943_2014_276_MOESM2_ESM.doc]

Additional file 2: Figure S1. RT-PCR of the transfection efficiency in GBC cell lines. (A) The transduction efficiency was determined at 3 days after infection with lentiviruses at a multiplicity of infection of 50. The transduced cells labeled with EGFP were observed under a fluorescence microscope. Light micrograph (upper); fluorescent micrograph (lower) (×100). (B) RT-PCR were performed to detect SPOCK1 expression in the indicated cells (**P*<0.05, ***P*<0.01, and ****P*<0.001).

**
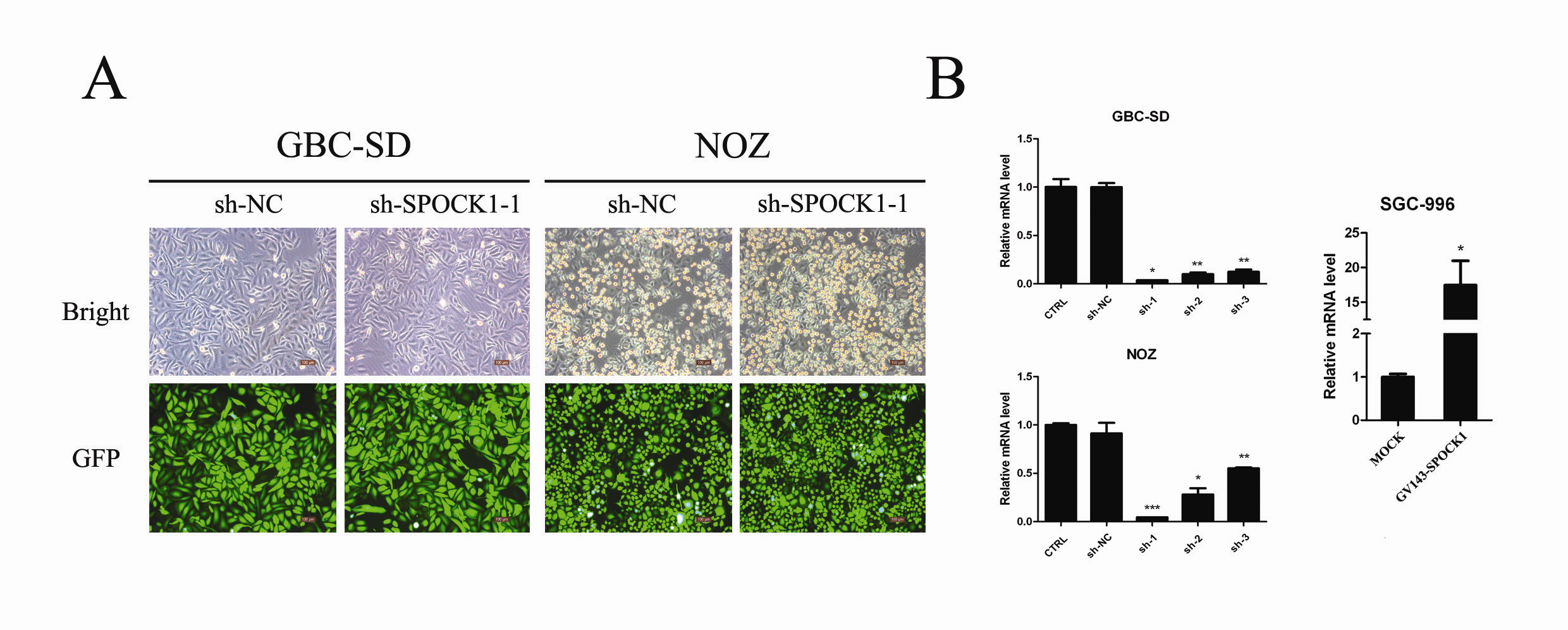
**
