# Supplementary material for: SPOCK1 as a potential cancer prognostic marker promotes the proliferation and metastasis of gallbladder cancer cells by activating the PI3K/AKT pathway
Source: Mol Cancer. 2015 Jan 27;14(1):12. doi: 10.1186/s12943-014-0276-y (PMC4320842; doi:10.1186/s12943-014-0276-y)

Additional file 3: Figure S2. Association of SPOCK1 expression on the growth of GBC cells *in vivo*. (A & B) Photograph of a subcutaneous xenograft model of human GBC in mice. (C) Immunohistochemical staining of Ki-67in tumor tissues of the subcutaneous xenograft model.


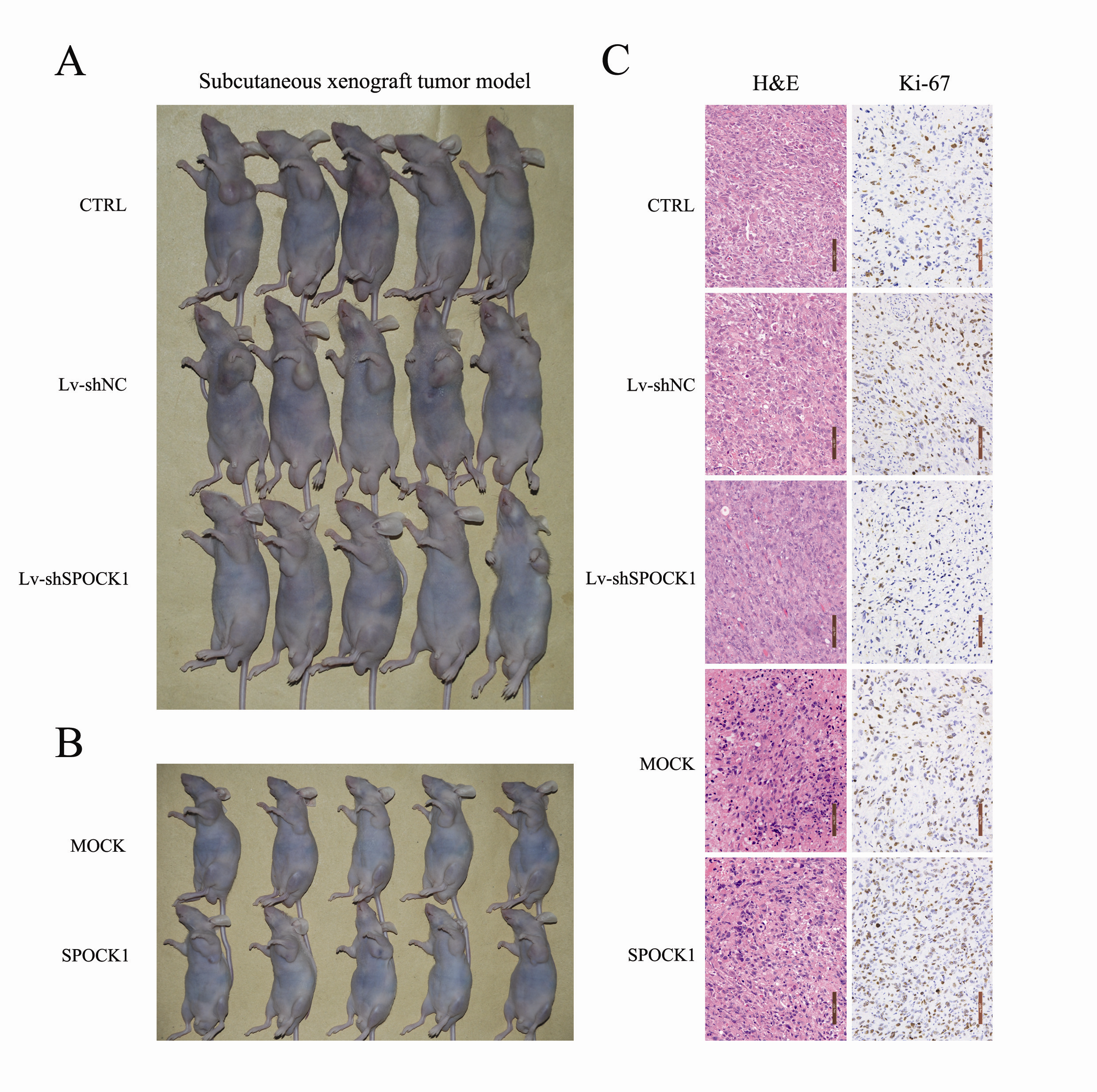

Supplement: Additional file 3: Figure S2. — Association of SPOCK1 expression on the growth of GBC cells in vivo. (A and B) Photograph of a subcutaneous xenograft model of human GBC in mice. (C) Immunohistochemical staining of Ki-67in tumor tissues of the subcutaneous xenograft model. [file 12943_2014_276_MOESM3_ESM.doc]
