# Supplementary material for: SPOCK1 as a potential cancer prognostic marker promotes the proliferation and metastasis of gallbladder cancer cells by activating the PI3K/AKT pathway
Source: Mol Cancer. 2015 Jan 27;14(1):12. doi: 10.1186/s12943-014-0276-y (PMC4320842; doi:10.1186/s12943-014-0276-y)

Additional file 4: Figure S3. SPOCK1 exerts an anti-apoptotic effect via the PI3K/Akt pathway in SGC-996 cells. (A) Apoptosis was determined in empty vector- and SPOCK1-transfected cells by flow cytometry. The apoptotic index was defined as the percentage of apoptotic cells. (B) The levels of phosphorylated PI3K (Tyr607), total PI3K, phosphorylated Akt (Ser473), total Akt were detected in empty vector- and SPOCK1-transfected cells by western blot analysis. GADPH was used as the loading control.


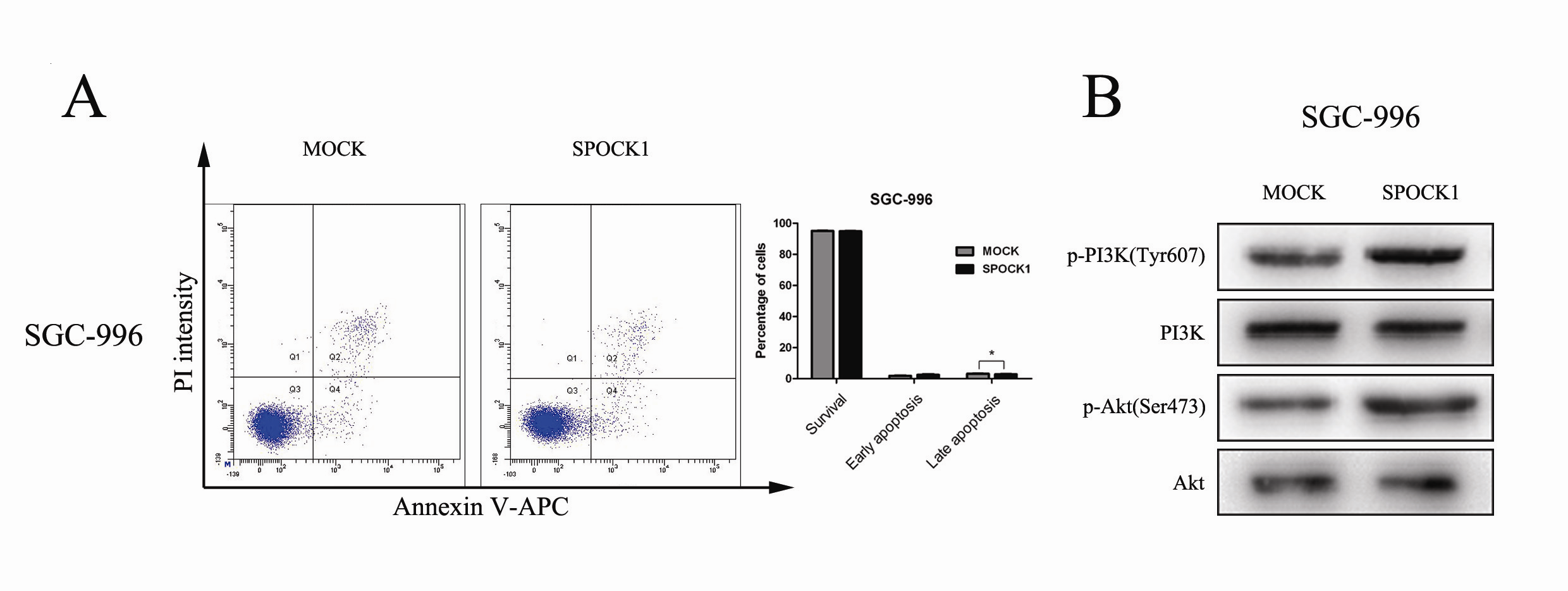

Supplement: Additional file 4: Figure S3. — SPOCK1 exerts an anti-apoptotic effect via the PI3K/Akt pathway in SGC-996 cells. (A) Apoptosis was determined in empty vector- and SPOCK1-transfected cells by flow cytometry. The apoptotic index was defined as the percentage of apoptotic cells. (B) The levels of phosphorylated PI3K (Tyr607), total PI3K, phosphorylated Akt (Ser473), total Akt were detected in empty vector- and SPOCK1-transfected cells by western blot analysis. GADPH was used as the loading control. [file 12943_2014_276_MOESM4_ESM.doc]
